# PTCy versus ATG as graft-versus-host disease prophylaxis in mismatched unrelated stem cell transplantation

**DOI:** 10.1038/s41408-024-01032-8

**Published:** 2024-03-15

**Authors:** Olaf Penack, Mouad Abouqateb, Christophe Peczynski, William Boreland, Zafer Gülbas, Tobias Gedde-Dahl, Cristina Castilla-Llorente, Nicolaus Kröger, Mathias Eder, Alessandro Rambaldi, Francesca Bonifazi, Igor Wolfgang Blau, Matthias Stelljes, Peter Dreger, Ivan Moiseev, Hélène Schoemans, Christian Koenecke, Zinaida Peric

**Affiliations:** 1 Medical Clinic, Department for Haematology, Oncology and Tumorimmunology, Charité Universitätsmedizin Berlin, Berlin, Germany; 2 EBMT Transplant Complications Working Party, Paris, France; 3 EBMT Paris Study Office, Department of Haematology, Saint Antoine Hospital, INSERM UMR-S 938, Sorbonne University, Paris, France; 4 Anadolu Medical Center Hospital, Kocaeli, Turkey; 5 Oslo University Hospital, Rikshospitalet, Oslo, Norway; 6 Gustave Roussy Cancer Campus, Villejuif, France; 7 University Medical Center, Department for Stem Cell Transplantation, Hamburg, Germany; 8 Hannover Medical School, Hannover, Germany; 9 ASST Papa Giovanni XXIII, Bergamo, Italy; 10 IRCCS Azienda Ospedaliero-Universitaria di Bologna, Bologna, Italy; 11 University of Muenster, Muenster, Germany; 12 University of Heidelberg, Heidelberg, Germany; 13 RM Gorbacheva Research Institute, Pavlov University, St Petersburg, Russia; 14 Department of Hematology, University Hospitals Leuven and KU Leuven, Leuven, Belgium; 15 Department of Haematology, University Hospital Centre Rijeka, Rijeka, Croatia

**Keywords:** Stem-cell research, Translational research, Graft-versus-host disease

## Abstract

There is an increased risk of GVHD and of non-relapse mortality (NRM) after allogeneic stem cell transplantations (alloSCT) when mismatched unrelated donors (MMUD) are used. In Europe, it is standard practice to use rabbit anti-thymocyte globulin (rATG) to reduce the high NRM and GVHD risks after MMUD alloSCT. As an alternative to rATG, post-transplantation Cyclophosphamide (PTCy) is in increasing clinical use. It is currently impossible to give general recommendations regarding preference for one method over another since comparative evidence from larger data sets is lacking. To improve the evidence base, we analyzed the outcome of rATG vs. PTCy prophylaxis in adult patients with hematologic malignancies undergoing first peripheral blood alloSCT from MMUD (9/10 antigen match) between Jan 2018 and June 2021 in the database of the European Society for Blood and Marrow Transplantation (EBMT). We performed multivariate analyses using the Cox proportional-hazards regression model. We included 2123 patients in the final analyses (PTCy, *n* = 583; rATG, *n* = 1540). *p* values and hazard ratios (HR) presented here are multivariate outcomes. Two years after alloSCT we found a lower NRM in the PTCy group of 18% vs. 24.9% in the rATG group; *p* = 0.028, HR 0.74. Overall survival in the PTCy cohort was higher with 65.7% vs. 55.7% in the rATG cohort; *p* < 0.001, HR 0.77. Progression-free survival was also better in the PTCy patients with 59.1% vs. 48.8% when using rATG; *p* = 0.001, 0.78. The incidences of chronic GVHD and acute GVHD were not significantly different between the groups. We found significantly lower NRM as well as higher survival in recipients of peripheral blood alloSCTs from MMUD receiving PTCy as compared to rATG. The results of the current analysis suggest an added value of PTCy as GVHD prophylaxis in MMUD alloSCT.

## Introduction

One of the main clinical challenges of allogeneic stem cell transplantation (alloSCT) is its inherent non-relapse mortality (NRM) with graft-versus-host disease (GVHD) as a major contributing factor. This problem is aggravated when mismatched unrelated stem cell donors (MMUD) are used, leading to specifically high NRM [1].

There is consensus in the field that patients after MMUD alloSCT should get an intensive GVHD prophylaxis regimen. It has been standard of care to use rabbit anti-thymocyte globulin (rATG, also termed anti-T-cell globulin or anti-T-lymphocyte globulin; products: Grafalon® or Thymoglobulin®) in alloSCTs from MMUD in Europe to decrease the GVHD and NRM risks. However, the prevention strategies of GVHD are currently changing. Cyclophosphamide given after alloSCT (post-transplant Cyclophosphamide, PTCy) is another option, which is increasingly used in some alloSCT centers.

Currently it is not possible to make sound evidence based decisions on the use of rATG or PTCy in MMUD alloSCT since comparative data from large data sets is missing. In addition, no randomized trials specifically compared PTCy vs. rATG prophylaxis in MMUD alloSCT. Previous smaller studies gave inconsistent results. A European Society for Blood and Marrow Transplantation (EBMT) matched control study suggested that PTCy could have advantages over rATG in the MMUD setting [2]. In the CTN 1703 and CTN 1203 randomized trials [3, 4], as well as in a comparative retrospective study [5], which showed benefit for PTCy, only a few (7/8) MMUD patients were enrolled. Two retrospective studies and a meta-analysis dedicated to MMUD alloSCT showed no significant reduction in the incidence or severity of aGVHD or cGVHD, in patients receiving PTCy, while a decreasing rate was estimated after adjusting for propensity [6–8]. The meta-analysis [7] highlighted a reduced NRM in the PTCy arm as compared to the rATG arm, which is in line with the results of the propensity-adjusted retrospective study [5]. However, in an EBMT retrospective cohort, the GVHD-free, relapse-free survival was not significantly different between PTCy and rATG [8]. Taken together the available evidence base is insufficient for clinical decision making.

To improve the evidence base, we analyzed outcomes of rATG vs. PTCy prophylaxis in adult patients with hematologic malignancies undergoing first peripheral blood alloSCT from 9/10 antigen MMUD between Jan 2018 and June 2021 in the database of the EBMT.

## Subjects and methods

### Study design and data collection

This is a retrospective multicenter analysis using the data set of the EBMT registry. The EBMT is a voluntary working group of more than 600 transplant centers which are required to report regular follow up on all consecutive stem cell transplantations. Audits are routinely performed to determine the accuracy of the data. The study was planned and approved by the Transplant Complications Working Party of the EBMT. All patients gave their written informed consent to use their personal information for research purposes. The study was conducted in accordance with the Declaration of Helsinki and Good Clinical Practice guidelines. Eligibility criteria for this analysis included patients older than 18 years of age at alloSCT with hematologic malignancies (acute lymphoblastic leukemia, acute myeloid leukemia, lymphoma, chronic lymphocytic leukemia, myelodysplastic syndrome or myeloproliferative neoplasms), who underwent a first alloSCT from a 9/10 antigen mismatched unrelated donor (MMUD), from a peripheral blood stem cells source, between Jan 2018 and June 2021 in the database of the EBMT. Only patients receiving either rATG or PTCy based GVHD prophylaxis (without a combined use of both) were included. Additionally, patients with more than one previous autologous transplantation, ex vivo T-cell depletion, or use of Alemtuzumab (Campath) were not included in the study. Data collected included recipient and donor characteristics (age, sex, cytomegalovirus serostatus and Karnofsky performance status score), diagnosis and status at transplant and transplant-related factors, including conditioning regimen, stem cell source and GVHD prophylaxis. GVHD grading was performed according to published criteria for acute GVHD [9] and chronic GVHD [10]. For the purpose of this study, all necessary data were collected according to the EBMT guidelines, using the EBMT Minimum Essential Data forms.

### Statistical analysis

Median values and interquartile ranges (IQR), and minimum and maximum values were used to describe quantitative variables; frequency and percentage were used for categorical variables. Main patient-, disease-, and transplant-related characteristics were compared using Pearson’s chi-squared or Fisher test for categorical variables, and the Kruskal–Wallis rank sum test for quantitative variables between the two groups.

Study endpoints were non-relapse mortality (NRM), overall survival (OS), progression-free survival (PFS), relapse incidence (RI), GVHD-free/relapse-free survival (GRFS), and incidence and severity of acute GVHD and chronic GVHD. The initial time was the date of transplant for all endpoints. NRM was defined as death without relapse/progression, PFS was defined as survival without relapse or progression, RI was defined as disease recurrence, GRFS was defined as survival without incidence of relapse, or grade III–IV acute GVHD, or severe chronic GVHD. Probabilities of OS, PFS and GRFS were calculated using the Kaplan-Meier method. Cumulative incidence was used to estimate NRM, RI, as well as acute and chronic GVHD in a competing risk setting, where death and relapse were considered as competing risks as appropriate [11]. Multivariate analyses were performed using the Cox cause-specific proportional-hazards model for all end points. All known potential risk factors, and variables differing significantly across the groups were included in the multivariate models: patient age at transplant, year of transplant, patient and donor gender, donor to patient CMV combination, Disease Risk Index (DRI), Karnofsky Performance Status (KPS), any level of total body irradiation (TBI), conditioning intensity (RIC vs. MAC). Center effect was taken into account by introducing a random effect or ‘frailty’ into all models. Results were expressed as the hazard ratio (HR) with the 95% confidence interval (95% CI). All tests were two-sided with a type 1 error rate fixed at 0.05. Statistical analyses were performed with R 4.3.0 software (R Development Core Team, Vienna, Austria) packages.

## Results

The baseline characteristics of the study population are presented in Table 1. A total of 2123 patients were included, from which 1540 (73%) received rATG, and 583 (27%) received PTCy as a GVHD prophylaxis.

**Table 1:**
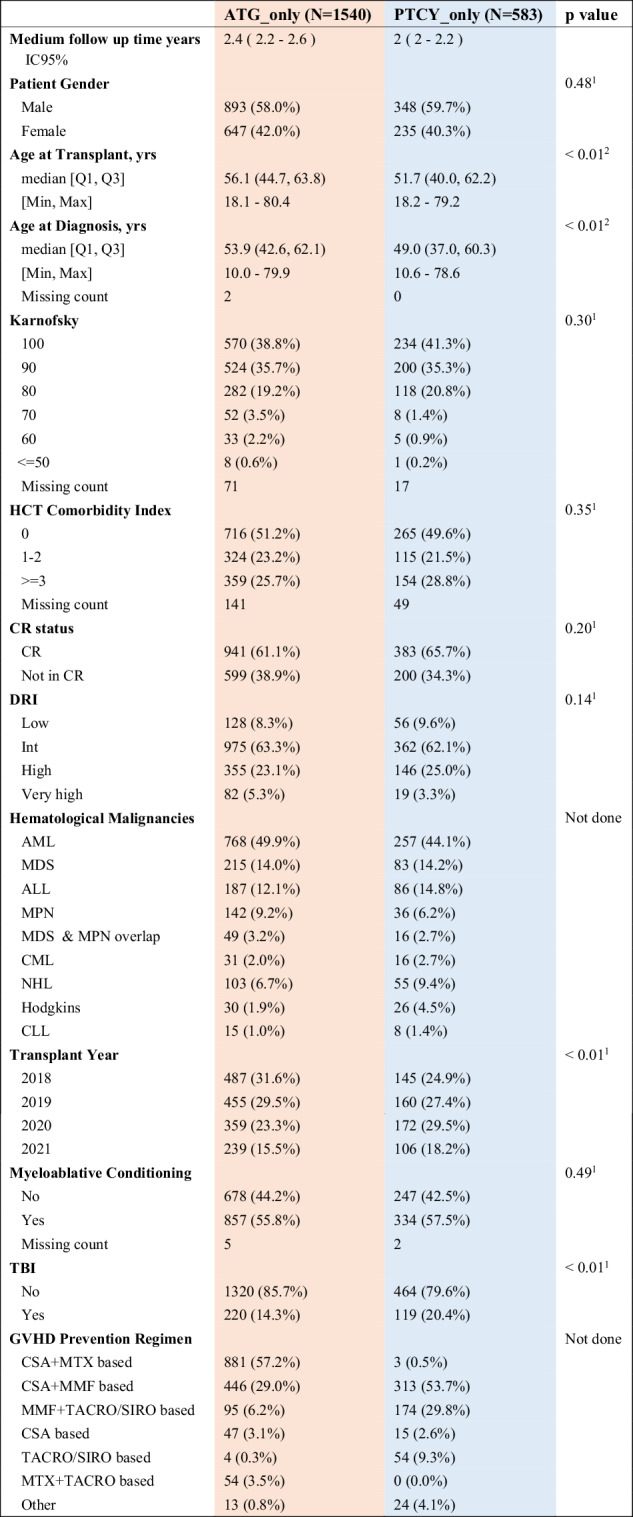
Baseline patient-, donor- and transplant-related characteristics by graft-versus-host disease prevention strategy.

Overall, the majority of patients was transplanted for acute leukemia (58.6%), myelodysplastic syndrome (MDS)/ myeloproliferative neoplasm (MPN) (25.6%) or lymphoma (15.1%). A high proportion of patients had a low/intermediate Disease Risk Index (DRI, 72%), and myeloablative conditioning (MAC) was more frequently performed (53.9%) than reduced intensity conditioning (RIC).

Patients in the rATG group were older, with a median age of 56.1 years (IQR 44.7, 63.8) vs. 51.7 years in the PTCy group (IQR 40.0, 62.2) (*p* ≤ 0.001), with a similar proportion of males (58% in rATG vs. 60% in PTCy, *p* = 0.48), and less recent transplants (*p* < 0.01), along with a significantly lower use of TBI (14.3% vs. 20.4%, *p* < 0.01). The remaining parameters were balanced between the two groups.

Median follow up was 2.0 years (95% CI [2–2.2]) in the PTCy arm and 2.4 years (95% CI [2.2–2.6]) in the rATG arm.

### Survival, RI and NRM

**Fig. 1:**
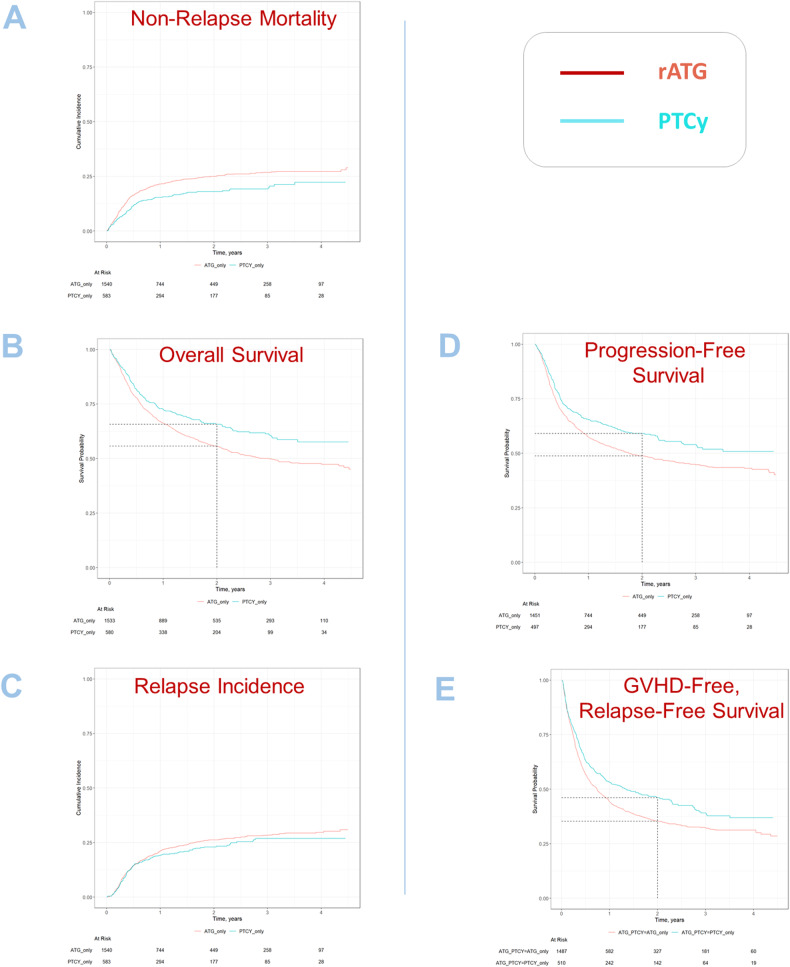
Survival outcomes and relapse. **A** Non-relapse mortality; **B** overall survival; **C** relapse incidence; **D** progression-free survival; **E** GVHD-free relapse-free survival.

Univariate outcomes are shown in Fig. 1 and Table 2. The results of the multivariate analyses are summarized in Table 3.

**Table 2:**
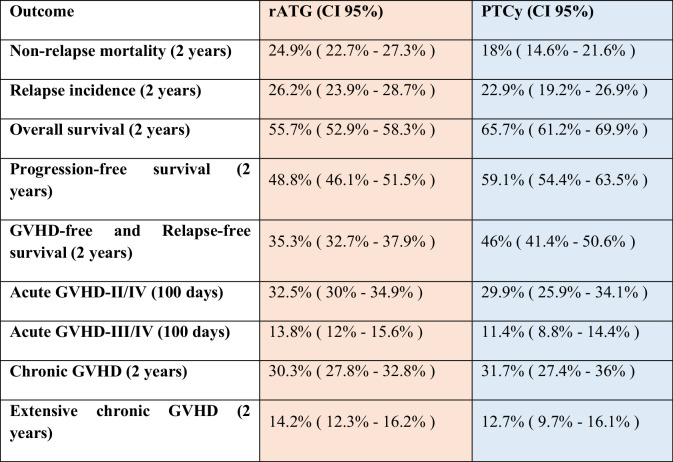
Incidence of univariate outcome parameters.

All parameters except acute GVHD are given at 2 years. Acute GVHD is given at day +100 after alloSCT. Numbers represent survival probability for the outcomes Overall survival, Progression-free survival, GVHD-free Relapse-free survival, and cumulative incidence for the outcomes Non-relapse mortality, Relapse incidence, Grades of Acute and Chronic GVHD.

**Table 3:**
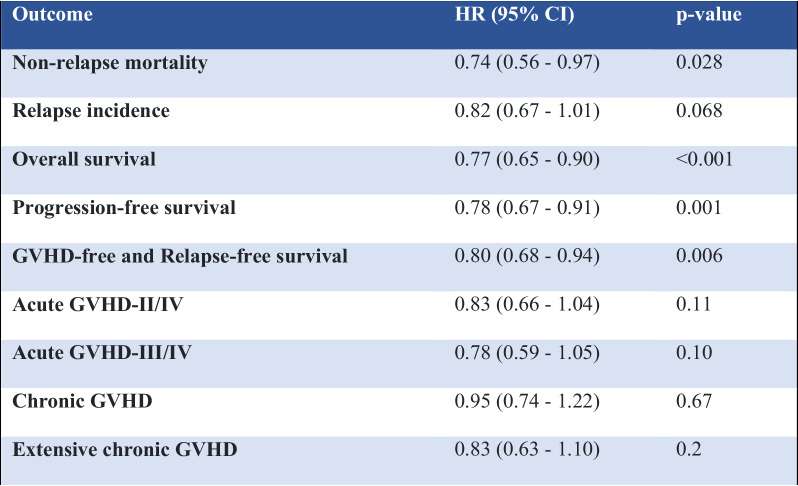
Multivariate Cox analysis: hazard ratios (HR) for PTCy vs. rATG, adjusted for potential risk factors and variables with significant difference across the groups.

Multivariate analysis. Hazard ratios (HR) are given for PTCy with rATG being the comparator^a^. The following variables were included in the multivariate models: Patient age at transplant, year of transplant, patient gender, donor gender, Donor to Patient CMV positivity, Disease Risk Index (DRI), Karnofsky Performance Status (KPS), total body irradiation (TBI), myeloablative conditioning (MAC), Center effect (frailty), intensity of conditioning and in vivo T-cell depletion. ^a^The provided adjusted hazard ratios (HR) are for PTCy relative to ATG. Each row represents an outcome from a separate fitted model. In addition to the primary prevention strategy comparison of PTCy vs. ATG, these models also adjust for the following factors: Patient age at transplant, year of transplant, patient gender, donor gender, Donor to Patient CMV positivity, Disease Risk Index (DRI), Karnofsky Performance Status (KPS), total body irradiation (TBI), myeloablative conditioning (MAC), Center effect frailty, intensity of conditioning.

Patients receiving PTCy had a significantly lower NRM as compared to patients receiving rATG (2 years incidence: 18% vs. 24.9%; HR: 0.74 [95% CI 0.56–0.97], *p* = 0.028). Similarly, OS and PFS showed a statistically significant and clinically meaningful benefit for PTCy arm, with a higher OS (2 years incidence: 65.7% vs. 55.7%; HR: 0.77 [95% CI 0.65–0.90], *p* < 0.001), and a higher PFS (2 years incidence: 59.1% vs. 48.8%; HR: 0.78 [95% CI 0.67–0.91], *p* = 0.001). RI did not differ significantly between the two groups, however, there was a trend toward a lower relapse rate in the PTCy arm vs. the rATG arm (2 years incidence: 22.9% vs. 26.2%; HR: 0.82 [95% CI 0.67–1.01], *p* = 0.068) (see Fig. 1).

Relapse of the underlying malignancy was the most frequent cause of death, accounting for 287 (44%) of total deaths in both arms, followed by NRM causes: infections (161 [19%]), GVHD (150 [18%]) and other alloSCT-related causes (150 [9%]) of total deaths. Secondary malignancies contributed to ~1% of total deaths, proportion for each arm are presented in Table 4.

**Table 4:**
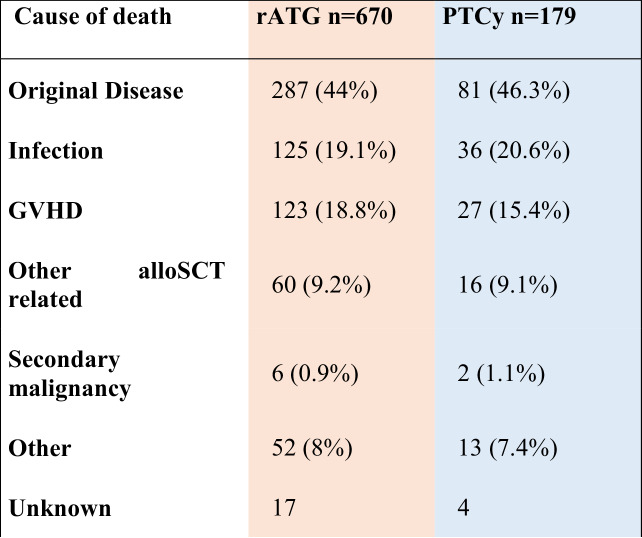
Distribution of causes of death in ATG-only versus PTCY-only regimens.

Numbers refer to frequencies and percentages within each group.

After conducting an additional multivariate cox analysis, adjusting for HLA mismatches location additionally to the previous factors, we found no hazard ratio (HR) for HLA mismatches to be statistically significant. This indicates that the differences in outcomes are not attributable to HLA-A, B, or C mismatches in our context. Furthermore, our results consistently demonstrate a significant benefit in favor of PTCy relatively to ATG. Specifically, we observed a notable reduction in non-relapse mortality (NRM) associated with PTCy compared to ATG, with a hazard ratio (HR) of 0.66 [CI 95%: (0.49–0.88); *p* value: 0.005], similarly there was a significant improvement in overall survival (OS) for PTCy relatively to ATG [HR: 0.69, CI 95%: (0.56–0.83); *p* value < 0.001], we also observed a reduced risk for progression-free survival (PFS) in PTCy relatively to ATG [HR: 0.71 CI 95%: (0.59–0.86); *p* value < 0.001]. These results were further supported by the fact that no significant interaction term between PTCy vs. ATG and Mismatch Location has been found, suggesting that the beneficial effect of PTCy over ATG is consistent across different HLA mismatch subgroups.

### Incidence of acute and chronic GVHD, and GRFS

**Fig. 2:**
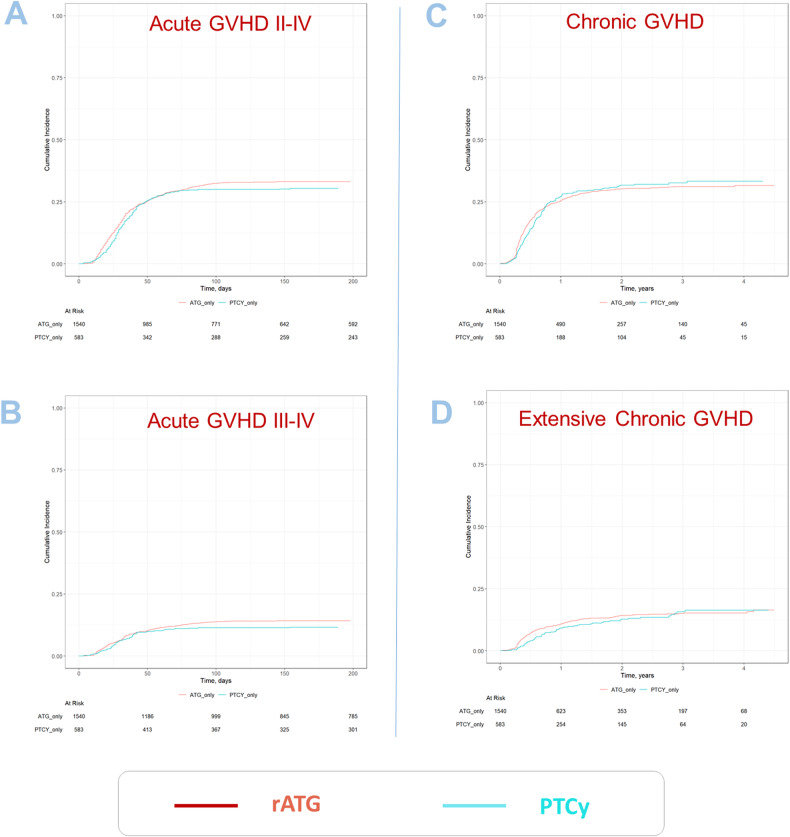
Cumulative incidences of GVHD. **A** Acute GVHD II–IV, **B** acute GVHD III–IV, **C** chronic GVHD all grades, **D** extensive chronic GVHD.

No significant difference was observed in acute and chronic GVHD outcomes between the two groups (see Fig. 2). The incidence of acute GVHD grades II–IV in patients receiving PTCy vs. rATG (100 days incidence: 29.9% vs. 32.5%; HR: 0.83 [95% CI 0.66–1.04], *p* = 0.11), and the incidence of severe acute GVHD grades III–IV (100 days incidence: 11.4% vs. 13.8%; HR: 0.78 [95% CI 0.59–1.05], *p* = 0.1), showed no significant difference. Similarly, overall chronic GVHD for patients receiving PTCy vs. rATG (2 years incidence: 31.7% vs. 30.3%; HR 0.95 [95% CI 0.74–1.22], *p* = 0.67), as well as extensive chronic GVHD (2 years incidence: 12.7% vs. 14.2%; HR 0.83 [95% CI 0.63–1.10], *p* = 0.20) were not statistically different.

GRFS was significantly higher in the PTCy arm compared to the rATG arm (2 years incidence: 46% vs. 35.3%; HR: 0.80 [95% 0.68–0.94], *p* = 0.006) (see Fig. 1).

## Discussion

In MMUD alloSCT, rATG or PTCy are readily used as part of the GVHD prophylaxis strategy. In Europe, it has been standard of care to use rATG in alloSCTs with a high GVHD risk [12], but there is to date no direct evidence available on PTCy prophylaxis vs. rATG use. Only a few (7/8) MMUD patients were enrolled in the CTN 1703 and CTN 1203 randomized trials [3, 4], as well as in a comparative retrospective study, suggesting efficacy of PTCy in the MMUD setting [5].

The results from the current study add to this emerging evidence by showing better outcomes of PTCy vs. rATG regarding NRM and survival after MMUD alloSCT in our real-world retrospective dataset. These data add to previous evidence comparing PTCy vs. rATG prophylaxis in MMUD alloSCT. One study found in patients with lymphoproliferative diseases undergoing 9/10 MMUD alloSCT a significantly lower extensive cGVHD rate when PTCy was used (PTCy 5% vs. ATG 18%) [13]. Another publication shows reduced aGVHD rates in MMUD alloSCT recipients when PTCy is used vs. ATG [14]. Jiminez et al. found Improved GRFS after PTCy vs. ATG-based MMUD alloSCT [15]. One smaller retrospective study demonstrated lower aGVHD and NRM rates in the PTCy arm without significant association to cGVHD [16]. Two retrospective studies and a meta-analysis showed no significant reduction in the incidence or severity of aGVHD or cGVHD after PTCy vs. rATG use in MMUD alloSCT, while a decreasing rate was estimated after adjusting for propensity [7, 8, 17]. A meta-analysis [7] highlighted a reduced NRM in the PTCy arm vs. rATG, win line with the results of the propensity-adjusted retrospective study [5] and with our current results. However, in another EBMT dataset report describing a smaller population of patients with lymphoma as underlying disease, GVHD-free, relapse-free survival was not significantly improved in a previous report from the EBMT [8].

The limitations of our current study are inherent to all retrospective real-world datasets, with low granularity, risk of underreporting and potential confounding factors. We also noted significant differences in baseline characteristics, with the rATG group being slightly older at diagnosis and transplantation, and having received more radiation therapy. The amount of missing data however was surprisingly low. Furthermore, because of the advent of PTCY prophylaxis is a relatively recent practice change, the observation times are still relatively limited, precluding conclusions regarding long-term outcome and the occurrence of late effects. For instance, we did not observe differences in secondary malignancies but long-term follow up will be needed to answer the question if PTCy has relevant carcinogen effects in this specific setting. We also noticed a wide variety of immunosuppressive regimens given alongside the rATG or PTCY prophylaxis, whose effect is, by design, difficult to tease out.

In the present study, we found a statistically non-significant trend toward a lower incidence of relapse in patients receiving PTCy vs. rATG. These data raises the question of whether patients with certain tumor entities benefit particularly strongly from PTCy use. Future studies will need to focus on the differential impact of PTCy vs. rATG on relapse rates in different tumor entities (e.g., lymphoid malignancies vs. myeloid neoplasms) led by disease specific working parties with access to large sets of patient data (e.g., EBMT or CIBMTR).

Taking together all the available evidence from the current study as well as from previous publications, it becomes evident that rATG and PTCy are both of clinical use in MMUD alloSCT. One of the possible next steps is to investigate the combination of both strategies to further increase efficacy in the MMUD setting [18]. A combination of rATG and PTCy has been tested by several investigators in haploidentical SCT (haploSCT) [19–21]. Gao et al. combined PTCy and rATG with tacrolimus in a single arm study in 67 haploSCT recipients and found a low incidence of severe acute GVHD [20]. Chen at al. compared the outcome after rATG/PTCy (*n* = 61) with historical data from patients undergoing haploSCT with sirolimus/PT-Cy prophylaxis and found similar aGVHD and cGVHD rates in both arms but a higher overall survival in the rATG/PTCy arm [19]. Zhang et al. published a randomized controlled trial where 122 haploSCT recipients were randomly assigned 1:1 to either a PTCy/ATG or a standard-dose ATG group (“Beijing Protocol”, ATG: 10 mg/kg) [21]. The cumulative incidence of grade II–IV acute GVHD was significantly lower in the PTCy/ATG group (11.5% vs. 39.3%). Furthermore, 2-year overall survival (75.4% vs. 54.1%) and disease-free survival (72.7% vs. 55.0%) were significantly improved in the PTCy/ATG group. In the setting of MMUD there is less data available on the combination of PTCy and ATG. Deotare et al. published their experience with a combination of PTCy and rATG in MUD (*n* = 22) and MMUD (*n* = 6) alloSCT comparing it to with 27 historical cohort patients receiving rATG [22]. The cumulative incidence of acute GVHD (17% vs. 33%) and severe grade III–IV aGVHD (7% vs. 25%) was significantly lower in the rATG/PTCy cohort but survival was not different. Xue et al. reported on a pilot study in 21 alloSCT recipients including 4 MMUD alloSCT where they added low dose rATG to their standard PTCy GVHD prophylaxis [23]. Using a matched-pair analysis with a historic control receiving PTCy only, they found significantly lower cGVHD incidence (15% vs. 41%) but no significant differences in aGVHD and in survival outcomes. In summary, there is currently not enough evidence to recommend a combination of rATG with PTCy in routine clinical use in MMUD alloSCT but considerable emerging data suggesting that this should be a focus area for clinical research.

In this study, we found significantly lower NRM as well as higher survival in recipients of peripheral blood alloSCTs from MMUD receiving PTCy compared to rATG. The results of the current analysis build on the available evidence suggesting a preferential use of PTCy as GVHD prophylaxis in MMUD alloSCT.

### Supplementary information

Supplemental Material

## Data Availability

Individual participant data will not be shared because patients agreed to data sharing with EBMT as well as with publication of results, but not to share data with third parties.
