# Supplementary material for: PTCy versus ATG as graft-versus-host disease prophylaxis in mismatched unrelated stem cell transplantation
Source: Blood Cancer J. 2024 Mar 15;14(1):45. doi: 10.1038/s41408-024-01032-8 (PMC10940681; doi:10.1038/s41408-024-01032-8)
Supplement: Supplementary file 1 — Supplemental Material [file 41408_2024_1032_MOESM1_ESM.docx]

|  | Table S1 Multivariable Cox regression analysis of survival and relapse related outcomes | | | | | | | | | |
| --- | --- | --- | --- | --- | --- | --- | --- | --- | --- | --- |
|  |  | **Non Relapse Mortality NRM** | **Relapse Incidence RI** | | **Overall Survival OS** | | **Progression Free Survival PFS** | | **GRFS** | |
| **Variable** |  | **HR** **(95% CI)***^1^*  **p-value***^2^* | | **HR** **(95% CI)***^1^*  **p-value***^2^* | | **HR** **(95% CI)***^1^*  **p-value***^2^* | | **HR** **(95% CI)***^1^*  **p-value***^2^* | **HR** **(95% CI)***^1^*  **p-value***^2^* |  |
| **ATG vs PTCy** | PTCy | 0.74 (0.56 - 0.97)  **0.028*** | | 0.82 (0.67 - 1.01)  **0.068** | | 0.77 (0.65 - 0.90)  **<0.001***** | | 0.78 (0.67 - 0.91)  **0.001**** | 0.80 (0.68 - 0.94)  **0.006**** |  |
| **Patient Sex** | Female | 0.75 (0.62 - 0.92)  **0.007**** | | 0.92 (0.76 - 1.11)  0.40 | | 0.83 (0.72 - 0.95)  **0.009**** | | 0.84 (0.73 - 0.96)  **0.010*** | 0.92 (0.81 - 1.04)  0.16 |  |
| **Donor Sex** | Female | 0.94 (0.75 - 1.16)  0.56 | | 1.05 (0.86 - 1.29)  0.61 | | 1.01 (0.87 - 1.18)  0.86 | | 1.00 (0.86 - 1.15)  0.95 | 1.05 (0.92 - 1.19)  0.47 |  |
| **Age at HSCT**, yrs |  | 1.02 (1.01 - 1.03)  **<0.001***** | | 1.00 (0.99 - 1.01)  0.74 | | 1.02 (1.01 - 1.02)  **<0.001***** | | 1.01 (1.00 - 1.02)  **<0.001***** | 1.01 (1.00 - 1.01)  **<0.001***** |  |
| **Karnofsky** | (KPS>= 90) | 0.82 (0.65 - 1.04)  0.10 | | 0.93 (0.75 - 1.15)  0.50 | | 0.82 (0.69 - 0.97)  **0.018*** | | 0.89 (0.76 - 1.04)  0.13 | 0.84 (0.73 - 0.97)  **0.019*** |  |
| **Disease Risk Index (DRI)**  (Ref : Low) | Int | 0.89 (0.63 - 1.27)  0.53 | | 1.54 (1.12 - 2.11)  **0.009**** | | 1.23 (0.96 - 1.57)  0.10 | | 1.20 (0.95 - 1.51)  0.12 | 1.16 (0.92 - 1.47)  0.21 |  |
|  | High & Very High | 1.16 (0.80 - 1.69)  0.44 | | 3.36 (2.36 - 4.79)  **<0.001***** | | 2.10 (1.61 - 2.75)  **<0.001***** | | 2.03 (1.57 - 2.62)  **<0.001***** | 1.60 (1.25 - 2.06)  **<0.001***** |  |
| **Year of transplant** |  | 0.98 (0.89 - 1.08)  0.64 | | 1.05 (0.96 - 1.15)  0.30 | | 1.02 (0.95 - 1.10)  0.51 | | 1.01 (0.95 - 1.08)  0.69 | 0.97 (0.92 - 1.03)  0.35 |  |
| **Donor -Patient CMV**  (Ref : Pos-Pos) | Pos - Neg | 0.86 (0.58 - 1.26)  0.43 | | 0.90 (0.64 - 1.27)  0.56 | | 0.85 (0.66 - 1.11)  0.23 | | 0.88 (0.69 - 1.13)  0.32 | 0.91 (0.72 - 1.15)  0.42 |  |
|  | Neg - Pos | 1.11 (0.88 - 1.40)  0.37 | | 1.21 (0.96 - 1.51)  0.11 | | 1.11 (0.94 - 1.32)  0.22 | | 1.15 (0.97 - 1.35)  0.10 | 1.08 (0.94 - 1.25)  0.28 |  |
|  | Neg - Neg | 0.83 (0.63 - 1.09)  0.17 | | 0.82 (0.64 - 1.05)  0.12 | | 0.78 (0.65 - 0.94)  **0.009**** | | 0.81 (0.68 - 0.97)  **0.021*** | 0.92 (0.78 - 1.08)  0.29 |  |
| **TBI** | Yes | 0.87 (0.63 - 1.19)  0.37 | | 0.93 (0.72 - 1.22)  0.62 | | 0.87 (0.71 - 1.06)  0.16 | | 0.91 (0.75 - 1.10)  0.31 | 0.93 (0.78 - 1.11)  0.43 |  |
| **Myeloablative Conditioning** | Yes | 1.00 (0.81 - 1.25)  0.97 | | 0.94 (0.77 - 1.15)  0.54 | | 1.05 (0.90 - 1.22)  0.54 | | 0.98 (0.85 - 1.13)  0.77 | 1.02 (0.89 - 1.17)  0.77 |  |
|  |  | | | | | | | | | |

|  | *Table S2 Multivariable Cox regression analysis of GVHD related outcomes* | | | | | |
| --- | --- | --- | --- | --- | --- | --- |
|  |  | **cGVHD** | **cGVHD EXT** | **aGVHD-II/IV** | | **aGVHD-III/IV** |
| **Variable** |  | **HR** **(95% CI)***^1^*  **p-value***^2^* | **HR** **(95% CI)***^1^*  **p-value***^2^* | **HR** **(95% CI)***^1^*  **p-value***^2^* | | **HR** **(95% CI)***^1^*  **p-value***^2^* |
| **ATG vs PTCy** | PTCy | 0.95 (0.74 - 1.22)  0.67 | 0.83 (0.63 - 1.10)  0.20 | 0.83 (0.66 - 1.04)  0.11 | | 0.78 (0.59 - 1.05)  0.10 |
| **Patient Sex** | Female | 0.95 (0.79 - 1.14)  0.55 | 0.98 (0.75 - 1.26)  0.86 | 1.09 (0.92 - 1.30)  0.31 | | 1.17 (0.90 - 1.52)  0.24 |
| **Donor Sex** | Female | 1.33 (1.10 - 1.61)  **0.003**** | 1.29 (0.97 - 1.70)  **0.076** | 0.94 (0.78 - 1.13)  0.52 | | 1.00 (0.76 - 1.33)  0.99 |
| **Age at HSCT**, yrs |  | 1.00 (1.00 - 1.01)  0.41 | 1.01 (1.00 - 1.02)  **0.043*** | 1.00 (0.99 - 1.00)  0.28 | | 1.00 (0.99 - 1.01)  0.77 |
| **Karnofsky** | (KPS>= 90) | 0.99 (0.79 - 1.23)  0.90 | 0.81 (0.60 - 1.10)  0.18 | 1.05 (0.85 - 1.29)  0.68 | | 0.95 (0.70 - 1.28)  0.72 |
| **Disease Risk Index (DRI)**  (Ref : Low) | Int | 1.03 (0.75 - 1.41)  0.87 | 1.01 (0.66 - 1.56)  0.96 | 0.82 (0.62 - 1.10)  0.18 | | 0.75 (0.47 - 1.20)  0.23 |
|  | High & Very High | 1.04 (0.74 - 1.48)  0.81 | 0.96 (0.59 - 1.56)  0.87 | 0.85 (0.62 - 1.15) | 0.29 | 0.76 (0.46 - 1.26)  0.29 |
| **Year of transplant** |  | 0.93 (0.85 - 1.02)  0.13 | 0.85 (0.75 - 0.97)  **0.013*** | 1.01 (0.93 - 1.09)  0.89 | | 1.01 (0.90 - 1.14)  0.84 |
| **Donor -Patient CMV**  (Ref : Pos-Pos) | Pos - Neg | 0.93 (0.67 - 1.28)  0.65 | 0.93 (0.59 - 1.49)  0.77 | 0.98 (0.71 - 1.34)  0.89 | | 1.24 (0.77 - 2.00)  0.38 |
|  | Neg - Pos | 0.77 (0.62 - 0.97)  **0.023*** | 1.08 (0.79 - 1.47)  0.64 | 0.89 (0.72 - 1.09)  0.27 | | 0.81 (0.60 - 1.10)  0.18 |
|  | Neg - Neg | 0.78 (0.61 - 1.00)  **0.050*** | 1.10 (0.79 - 1.54)  0.57 | 1.10 (0.88 - 1.37)  0.40 | | 1.19 (0.84 - 1.68)  0.32 |
| **TBI** | Yes | 1.23 (0.95 - 1.59)  0.12 | 0.99 (0.69 - 1.41)  0.94 | 1.06 (0.83 - 1.36)  0.64 | | 0.98 (0.68 - 1.43)  0.92 |
| **Myeloablative Conditioning** | Yes | 0.82 (0.66 - 1.01)  **0.059** | 1.03 (0.78 - 1.36)  0.85 | 0.83 (0.68 - 1.00)  **0.052** | | 0.81 (0.61 - 1.06)  0.13 |

| *^1^* HR = Hazard Ratio, CI = Confidence Interval |
| --- |
| *^2^* *p<0.05; **p<0.01; ***p<0.001 |
